# Transactions of the Texas State Medical Association

**Published:** 1887-02

**Authors:** 


### Book Reviews.

TRANSACTIONS OF THE TEXAS STATE MEDICAL ASSOCIATION. Eighteenth Annual Session, held at Dallas, Texas, April 27, 28, 29 and 30, 1886. Austin, Texas. Printed for the Texas State Medical Association. 1886.

By our notice of the *Transactions* for 1885 in the issue of THE JOURNAL for July, 1886, it will be observed that "the correspondence of this volume in all its material characteristics to the tasteful and artistic appearance of the *Transactions of the Medical Association of Georgia* for past years indicates an appreciation on the part of our Texas brethren of the Georgia model, which should gratify any member of our body. With the Lone Star in place of the clasped hands and the black instead of the green cover, there is scarcely another feature by which to distinguish the Texas from the Georgia volume of Transactions."

As our brother Daniel had occasion to feel much flattered that we had adopted almost precisely his style of cover and arrangement of contents, from his failue to note the fact that Georgia took the lead in both enterprises, we again have occasion to congratulate Texas upon following this good example and improving on it by the heading of the pages, but detracting by an advertisement on the back of the title-page which mars the elegance of the volume. The size of the book is nearly double that of ours, owing to ample funds rather than abundant material. The Texas profession occupies a most enviable position in having 454 representatives in the State Association, and receipts amounting to $2,071.81, leaving, after a most liberal allowance for expenditures, a balance in the treasury.

Not only is a salary of $200 given the Secretary, but his expenses of all kinds are paid, and the extra duties on the Publishing Committee received a compensation of $200, while the Treasurer is paid a salary of $150. Independent of these provisions, $100 is set apart for a prize essay to encourage original investigations. These are very commendable features in the business department of the Association, and we are pleased to note that the literary and scientific work of this body compares favorably with the productions of other medical organizations throughout this country.

In the section of practice, Dr. S. H. Stout contributes a timely article on the prophylaxis of small-pox; and while his recommendation of humanized vaccine in preference to the bovine virus is based upon observation of their relative effects, we are at a loss to understand his objection to the vaccine farms which have been established in different sections, unless he can discover some other mode of getting fresh supplies of virus.

The English population in Great Britain and in Canada have become prejudiced against vaccination on account of its contamination with other diseases in the human subject, and our own people share this feeling, so that it becomes a choice of evils to use bovine virus.

In the section of dermatology it is a matter of surprise that nothing appears excepting a general review of the subject by the chairman, Dr. F. E. Daniel. After touching upon the rather tardy development of ascertained principles in skin nasalogy, he claims that the crying need of the hour is a new and simple classification—a revision of the nomenclature and the adoption of a new one, say by the National Congress—which, being based upon the part, or structure, or tissue of the skin affected, shall be accepted as the standard. What is wanted in dermatology is a better knowledge of known diseases rather than the discovery of unknown types.

Of the numerous practical papers embodied in this book of 691 pages, we have only space to express our conviction of the mutual benefit derived by the members from such contributions on a great variety of subjects by different observers, while those outside, who are so fortunate as to be found with this well-assorted report, will certainly relish the savory dishes that make up the feast of good things.

We have to lament the loss of the former efficient Secretary by the death of Dr. W. J. Burt, but his mantle could not have fallen upon one more worthy to bear it than on Dr. F. E. Daniel, who has shown himself equal to whatever he undertakes in business as well as in literary enterprises.

